# Effect of perforated low-density polyethylene films on postharvest quality of avocado fruit

**DOI:** 10.1016/j.heliyon.2024.e27656

**Published:** 2024-03-06

**Authors:** Nana Millicent Duduzile Buthelezi, Tieho Paulus Mafeo

**Affiliations:** a Department of Biology and Environmental Sciences, Sefako Makgatho Health Sciences University, P.O. Box 235, Medunsa, 0204, Ga-Rankuwa, South Africa; b Department of Plant Production, Soil Science and Agriculture and Engineering, School of Agricultural and Environmental Sciences, Faculty of Science and Agriculture, University of Limpopo, Private Bag X1106, 0727, Sovenga, South Africa

**Keywords:** Decay, Firmness, Lipoxygenase activity, Packaging films, Postharvest quality, Shelf life

## Abstract

Avocado (*Persea americana* Mill.) being a climacteric fruit, is very prone to quality deterioration and spoilage due to high metabolic activities which leads to postharvest and economic losses. The purpose of this study was to evaluate the impact of perforated low-density polyethylene (LDPE) packaging films on postharvest quality and shelf life of ‘Fuerte’ avocado. Fruit were packaged in LDPE plastics (20 and 40 μm) whereas unpackaged fruit were considered as control. Fruit were kept at ambient environments (21 ± 1 °C and 60.0 ± 5% RH) for 12 days and sampled at 4 days interval. The in-pack avocado created a suitable headspace with low O_2_ and high CO_2_ concentrations, which yielded improved preservation of postharvest quality and prolonged shelf life of the avocado. Fruit packed in both 20 and 40 μm LDPE films had lower ethylene production and respiration rates, weight loss, firmness loss, preserved fruit size, high pH, titratable acidity, low soluble solid content, sugar:acid ratio, malondialdehyde content and lipoxygenase activity compared to control. Fruit in LDPE films had no symptoms of decay (20 μm) and slight incidence and decay (40 μm) and were markable during shelf life compared to control fruit had severe decay symptoms and were unmarkable at the end of shelf life. These findings indicated that LDPE films were effective in preserving postharvest quality and extending shelf life of avocado fruit.

## 1 Introduction

Avocado (*Persea americana* Mill.) is highly ranked as an economically significant tropical and climacteric fruit worldwide, with an estimated yearly production of over 6.40 million metric tons, equalling 12.90 billion US Dollars, a 23.00% increase from 2017 compared to the global production of about 5.20 million metric tons prior to 2017 [1,2]. The important economic impact, high nutritional value and exceptional flavour make avocado a valuable fruit with high consumer demand [3]. It is predominantly rich in oleic acid, vitamin C, E, K and B_2_, magnesium and potassium among others [2]. These health promoting phytochemicals aid in lowering the occurrence of cardiovascular conditions, elevated cholesterol levels, diabetes and obesity [1,4]. Although avocado has significant economic importance, it is prone to perishability due to its physiological climacteric nature, resulting in a short shelf life of less than five weeks when kept at ideal storage conditions [1,4,5].

Avocado experiences a peak in climacteric activity following harvest and before it ripens. During this time, there is a significant increase in cellular respiration, ethylene synthesis and polygalacturonases and pectinases enzyme activities [6]. This climacteric peak result in fruit softening, increase in respiration rate and moisture loss through transpiration which causes mass loss [7], consequently, avocado ripens and spoils rapidly [5]. Furthermore, extreme fruit moisture loss, besides causing loss of marketable weight, stimulates browning, loss of firmness and flavour, accelerated senescence, proneness to chilling injury and membrane disintegration [8]. This results in major economic losses as the price of fresh fruit is mostly evaluated by its mass [5]. Therefore, any postharvest treatment that reduces fruit quality degradation by decreasing moisture loss and preserving fruit turgidity and texture, may possibly decrease respiration and ripening of the fruit, thus, prolonging it shelf life. Moreover, low-density polyethylene (LDPE) packaging films are an alternative postharvest technique for maintaining fruit quality and extending the shelf life of avocado [9].

Polyethylene is a synthetic polymer made of long-chain monomers of ethylene and is the most commonly used polymer film for packaging fruits and vegetables [9,10]. It is the simplest and the most inexpensive packaging plastic film [11]. Micro-perforated LDPE packaging film provide an excellent hermetic seal and gas exchange rates such as oxygen (O_2_) and carbon dioxide (CO_2_) to create the desired modified atmosphere and prevent water condensation, thus, improving quality and shelf life of various produce [12,13]. Perforated packaging is successfully used to prevent anaerobiosis, regulate water vapour, O_2_ and CO_2_, thereby reducing respiration rate and metabolic activity hence enhancing the bioactive compound characteristics and overall quality of perishable produce with high respiration rates during the storage period [14]. In addition, perforated LDPE packaging film could delay the ethylene process which stimulates fruit ripening and enzyme polyphenol oxidase activity, which causes browning of avocado fruit [14,15]. Limited information is available on the impact of LDPE materials on the overall quality and shelf of avocado stored at ambient conditions. The current study was therefore conducted to evaluate the efficacy of perforated LDPE packaging films on postharvest quality and shelf life of avocado.

## 2 Material and methods

### 2.1 Fruit sampling

Avocado fruit ‘Fuerte’ used in the current study were harvested from ZZ2 farm, Mooketsi, Limpopo, South Africa (23° 57′ 9.5″ S, 30° 14′ 3.5″ E). About 249 avocados with an average of 200–240 g per fruit were harvested thrice (making a total of 747 fruit) and packed in open-top boxes. Harvested fruit were then transported in a well-ventilated vehicle to Postharvest Laboratory of the University of Limpopo (25° 36′ 54″ S, 28° 0′ 59.76″ E) and later transported to Botany Laboratory of the Sefako Makgatho Health Science University, Pretoria, South Africa (25° 39′ 50.6″ S, 28° 08′ 01.1” E) for further analysis.

### 2.2 Postharvest treatment

When the fruit reached the Postharvest Laboratory, they were assigned to postharvest treatments of two LDPE packaging film; 20 and 40 μm thickness with the size of 200 × 330 mm (LDPE, Gundle Plastics Group, Johannesburg, South Africa). In a preliminary study, 20 and 40 μm LDPE films showed the best results compared to 60 and 80 μm (data not shown), thus 20 and 40 μm films were used in the current study. Fruit that were not packaged were used as control. The LDPE packaging films had perforations of 150 μm diameter from the top to the bottom of bags in two vertical rows. A total of 498 fruit without any visible defects per treatment were randomly selected and packed in perforated LDPE packaging films (accounting for 1 kg/packaging film) and 249 control fruit were used to conduct the research experiment. Generally, each treatment consisted of 3 replicates of 83 fruit per replicate. Fruit were subjected to ambient conditions at ± 21 °C and 60% RH, simulating retail or market conditions in the laboratory. Fruit chemical and sensory data was collected at 4 days intervals during the 12 days of shelf life.

### 2.3 Determination of gas analysis inside LDPE films

Gas composition inside the LDPE films was assessed with a portable gas analyzer (CO_2_/O_2_ gas analyzer, Model Q2, Quantek Instruments, Inc., Grafton, United States). The gas analysis was conducted by inserting a needle attached to the gas analyzer through an adhesive seal fixed on the lidding material [16]. The changes in the composition of respiratory gases (O_2_ and CO_2_) in sealed LDPE bags were measured during shelf life at 4 days interval. Gases for ambient atmosphere were used as control.

### 2.4 Determination of fruit ethylene production and respiration rate

Fruit ethylene production was determined using a handheld ethylene analyzer (F-950 three gas analyzer, Felix instruments, Camas, USA) [16]. Individual fruit were sealed in a 1 L jar for 15 min and the ethylene content was calculated and expressed as kg^−1^ h^−1^. Fruit respiration rate was determined with a gas analyzer (CO_2_/O_2_ gas analyzer, Model Q2, Quantek Instruments, Inc., Grafton, United States) at 4 days interval [5,16]. Individual fruit were sealed in a 1 L jar for 10 min, then the headspace CO_2_ concentration was measured and the results were expressed as mg kg^−1^ h^−1^, considering the fruit weight, headspace and ambient room CO_2_ concentration [5].

### 2.5 Determination of weight loss

Weight loss was assessed according to Shah and Hashmi [17] using an electronic weighing scale (HCB 1002, Adam equipment, Shanghai, China). The percentage of fruit weight loss was calculated using Eq. (1).

(1) $$\text{Weight}\;\text{loss}\;\left(\%\right)=\frac{\text{Initial}\;\text{fruit}\;\text{weight}-\text{Final}\;\text{fruit}\;\text{weight}}{\text{Initial}\;\text{fruit}\;\text{weight}}\times 100$$

### 2.6 Determination of firmness

Fruit firmness was evaluated based on a method of Tesfay and Magwaza [5], with minor modifications. Firmness was determined with a hand-held penetrometer (FT 327, Shalom Laboratory Supplies, Durban, South Africa) fitted with 8 mm spherical probe. Three firmness measurements were taken in the equatorial region of each fruit and the results were presented in newton (N).

### 2.7 Determination of fruit size

The size of the same individual fruit was measured during 12 days of shelf life with a digital electronic carbon fiber vernier caliper gauge micromete (150 mm LCD, Kawasaki, Japan) and the results were presented in mm [18].

### 2.8 Determination of pH, titratable acidity, soluble solid content and sugar:acid ratio

The soluble solid content (SSC) was measured according to Shah and Hashmi [17], with minor modifications. A 100 g of diced avocado fruit was weighed into a test tube containing 100 mL of distilled water. The mixture was then homogenized using a blender (Milex Nutri1200 8-In-1 Nutritional Blender, MNM003BK, Milex/Makro, Johannesburg, South Africa) for 1 min. The homogenate was filtered through a Miracloth® and the filtrate was centrifuged with a benchtop centrifuge (Rotina 380R, Labotec (PTY) LTD, Johannesburg, South Africa) at 20 000×*g* for 10 min. The supernatant was then used to measure the pH using a benchtop pH meter (PH-210, Masiye Labs, Johannesburg, South Africa) [19] and the SSC with a hand-held digital refractometer (Model 3810, ATAGO, PAL-1, Shalom Laboratory Supplies, Durban, South Africa) and the results were presented as °Brix (%).

The titratable acidity (TTA) was measured using a method of Nawab et al. [20], with some modifications. A 10 mL of homogenate was mixed with 0.3 mL of 1% phenolphthalein in 95% ethanol and titrated with 0.1 N sodium hydroxide to a permanent pink color (pH 8.1). The TTA was calculated using Eq. (2). A conversion factor of 0.28 was selected based on linoleic acid, a predominant acid in avocados [9].

(2) $$\text{TTA}=\frac{\left(0.1\times NaOH\times 0.28\times 1000\right)}{S}$$

where TTA is the titratable acid, 0.1 is 0.1 mol of NaOH [N], NaOH is the amount of NaOH added [mL], 0.28 is the conversion factor and S is the juice sample [mL].

Sugar:acid ratio was determined according to Al-Dairi [21] and calculated using Eq. (3) as follows:

(3) $$SSC:TTA\;\left(\%\right)\;\frac{SSC\;\left(\%\right)}{TTA\;\left(\%\right)}$$

where SSC is soluble solid content and TTA is titratable acid.

### 2.9 Determination of oxidative stress: malondialdehyde content and lipoxygenase activity

Malondialdehyde (MDA) content was determined based on the method described by Rasouli et al. [22], with minor modifications. A 3 g of avocado sample was mixed with 15 mL of 10% trichloroacetic acid and centrifuged (Hermle Labortechnik, Germany) at 20 000×*g* for 20 min. Thereafter, 1 mL of supernatant was mixed with 3 mL of 0.5% 2-thiobarbituric acid heated in a thermostatic water bath (DK-2000-IIIL, HINOTEK, Ningbo, China) at 90 °C for 30 min. Subsequently, the mixture was immediately allowed to cool on ice for 15 min and then centrifuged at 20 000×*g* for 15 min. The absorbance of each sample was read at 532 and 600 nm with a spectrophotometer (UV 1600 PC, Shimadzu, Milan, Italy). Lipid peroxidation was determined as total MDA equivalent (nmol g^−1^) and calculated as follows:

(4) $$\text{Total}\;\text{MDA}=\left[\frac{A_{532}\;nm\;-\;A_{600\;nm}}{155}\right]\times 1000$$

where A_532_ nm and A_600_ nm were an absorbance at 532 and 600 nm.

Lipoxygenase (LOX) activity was measured using the method of Li et al. [23], with minor alterations. Briefly, a mixture was prepared by combining 1 g of frozen mesocarp tissue powder with 3 mL of extraction buffer. The extraction buffer consisted of 50 mM potassium phosphate buffer at a pH of 7.80, 1 mM sodium-EDTA at a pH of 7.00 and 2% PVPP. The reaction mixture comprised of 0.09 M sodium phosphate buffer (pH 6), 0.17 mM linoleic acid sodium salt and 50 μL of crude enzyme extract in a final volume of 1.5 mL. The LOX activity was measured spectrophotometrically by recording the formation of hydroperoxides and the resulting increase in absorbance at 234 nm. The LOX activity was presented as the specific rate on a fresh weight basis of molar change of hydroperoxides in mmol kg^−1^ s^−1^.

### 2.10 Determination of fruit decay evaluation and the percentage of marketability

A randomized set of ‘Fuerte’ avocado fruit were evaluated for decay incidence according to Saidi et al. [24], with some alterations. Fruit decay incidence was assessed by trained panelists (15 males and 15 females) with the average age of 20–40 years at the Sefako Makgatho Health Sciences University (SMU), Department of Biology and Environmental Sciences laboratory at the end of shelf life. Stem end rot, side rot (alternaria rot and anthracnose) and overall decay were evaluated during shelf life of 12 days by assessing the fruit stem end or side decay. Decay was assessed on a 1–5 hedonic scale where 1 = no decay, 2 = slight decay, 3 = moderate decay, 4 = moderately severe decay and 5 = severe decay [25].

Marketability of the avocado fruit was rated according to a method of Saidi et al. [24], with some alterations. The descriptive quality characteristics were evaluated by assessing decay level, surface defects and shrinkage. A 1–9 score, with 1–3 = unmarketable, 4 = reasonable, 5–7 = good and 8–9 = excellent, was employed to assess the quality of the fruit. Fruit obtaining a rating of ≥5 were regarded marketable, whereas those rated <5 were regarded unmarketable. The quantity of saleable fruit was utilized as a metric for determining the percentage of marketable fruit during shelf life.

### 2.11 Statistical analysis

The collected data were subjected to analysis of variance (ANOVA) using GenStat statistical software GenStat®, 18.1 edition, VSN International, UK. Mean separation was performed using Fischer's least significant difference (LSD) at 5% level of significance. Standard error values were calculated where a significant standard deviation was found at *p* < 0.05 between individual values.

## 3 Results and discussion

### 3.1 Gas changes inside LDPE films

**Fig. 1:**
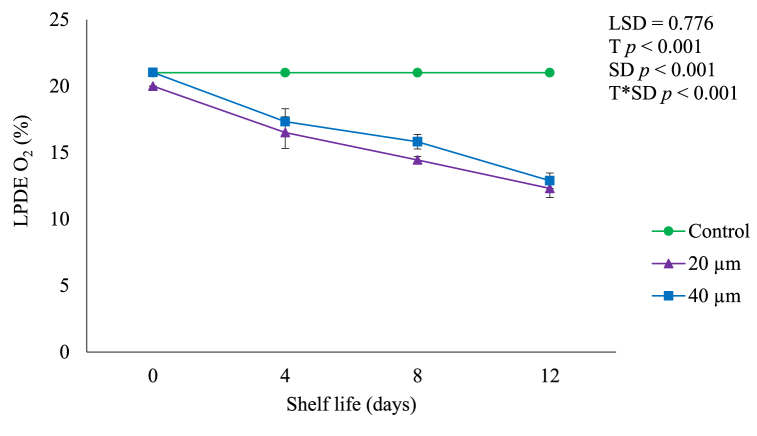
In pack gas composition in terms of O_2_ depletion in LDPE avocado package during shelf life of 12 days. Vertical bars represent standard error (SE) of the mean value (*n* = 3). The SE refers to the standard deviation of each measured values from the mean value. Means followed by different letters in each bar indicate a statistically significant difference (*p* < 0.001). LSD, least significant difference; T, treatments; SD, shelf life days; T*SD, treatments and shelf life days; LDPE, low-density polyethylene; O_2_, oxygen.

**Fig. 2:**
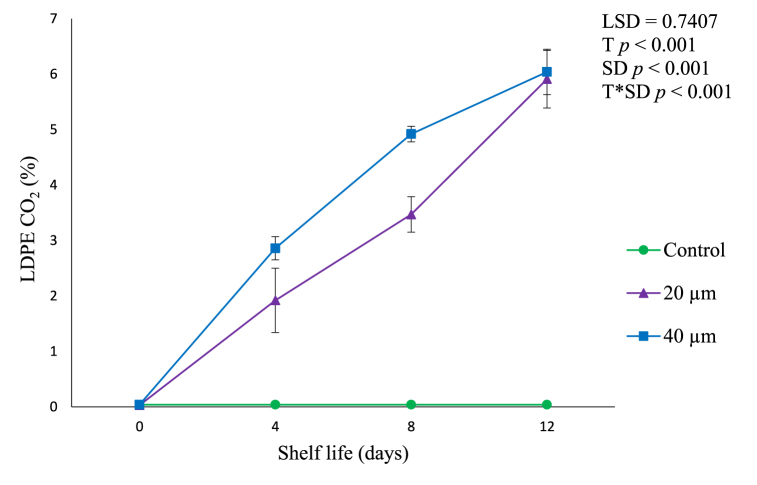
In pack gas composition in terms of CO_2_ accumulation in LDPE avocado package during shelf life of 12 days. Vertical bars represent standard error (SE) of the mean value (n = 3). The SE refers to the standard deviation of each measured values from the mean value. Means followed by different letters in each bar indicate a statistically significant difference (*p* < 0.001). LSD, least significant difference; T, treatments; SD, shelf life days; T*SD, treatments and shelf life days; LDPE, low-density polyethylene; CO_2_, carbon dioxide.

Due to processes such as product respiration, the gas composition within the package may inevitably undergo alterations [16]. During shelf life of 12 days, O_2_ in LDPE packaging plastics gradually (*p* < 0.05) decreased (Fig. 1) whereas CO_2_ significantly (*p* < 0.001) increased (Fig. 2). The O_2_ in 20 μm films decreased from 21.00 to 12.31% whereas CO_2_ increased from 0.03 to 5.91% during shelf life. Similarly, 40 μm films showed a decreasing O_2_ (21.03–12.89%) and increasing CO_2_ (0.041–6.04%) during shelf life. As expected, O_2_ (21.01%) and CO_2_ (0.04%) remained the same at ambient conditions. Decreased O_2_ and elevated CO_2_ levels in LDPE films could be created due to the process of respiration in produce [17]. Furthermore, reduced O_2_ and increased CO_2_ in the LDPE films can decrease ethylene production and sensitivity, leading to delayed fruit ripening [3,5].

### 3.2 Fruit ethylene production and respiration rate

**Fig. 3:**
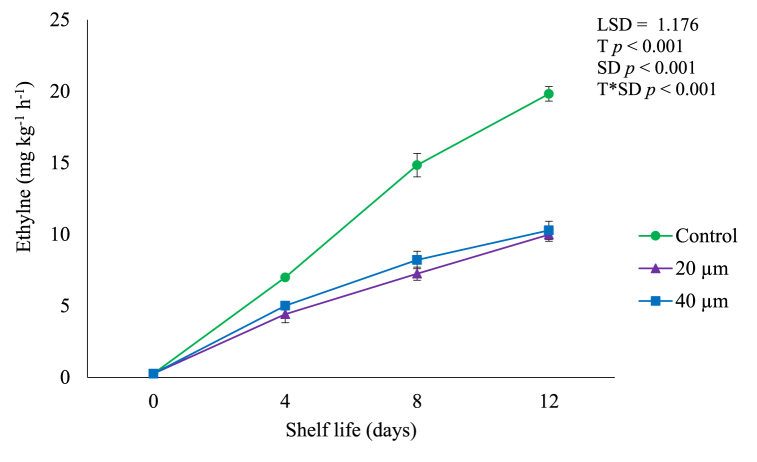
Ethylene production of avocado fruit packed in LDPE films during shelf life of 12 days. Vertical bars represent standard error (SE) of the mean value (*n* = 3). The SE refers to the standard deviation of each measured values from the mean value. Means followed by different letters in each bar indicate a statistically significant difference (*p* < 0.001). LSD, least significant difference; T, treatments; SD, shelf life days; T*SD, treatments and shelf life days.

Ethylene production rate of avocado fruit significantly (*p* < 0.05) increased with increasing shelf life (Fig. 3) due to the rate of metabolic reactions in fruit tissues [1,6,16]. However, LDPE films effectively (*p* < 0.05) reduced the rate of ethylene production in fruit than control. Fruit packaged in 20 and 40 μm films had significantly (*p* < 0.05) the lowest ethylene levels of up to 9.98 and 10.21 mg kg^−1^ h^−1^, respectively at the end of shelf life in comparison to control (19.82 mg kg^−1^ h^−1^). This shows that LDPE effectively suppressed metabolic reactions and reduced respiration rate. Reduced ethylene production rate in fruit packed in LDPE films could be attributed to decreased O_2_ (Fig. 1) which is a substrate for ethylene production, thus decreased O_2_ levels leads to less ethylene production [3,6]. Also, increased CO_2_ (Fig. 2) in LDPE films prevents ethylene production [5,12].

**Fig. 4:**
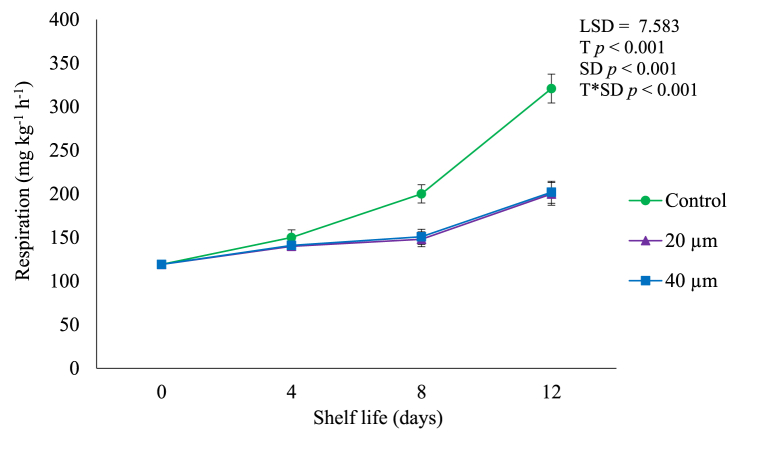
Respiration rate of avocado fruit packed in LDPE films during shelf life of 12 days. Vertical bars represent standard error (SE) of the mean value (*n* = 3). The SE refers to the standard deviation of each measured values from the mean value. Means followed by different letters in each bar indicate a statistically significant difference (*p* < 0.001). LSD, least significant difference; T, treatments; SD, shelf life days; T*SD, treatments and shelf life days.

In our study, a gradual (*p* < 0.05) rise in the rate of respiration was observed during shelf life of 12 days at ambient conditions (Fig. 4), implying that the respiration rate of avocado fruit increases as the fruit ripens or undergoes senescence [13,17]. However, LDPE films effectively (*p* < 0.05) reduced fruit respiration rate. At the end of shelf life, avocado fruit packed in 20 and 40 μm films had the lowest respiration rate of up to 200.01 and 201.04 mg kg^−1^ h^−1^, respectively whereas control had the high respiration rate of up to 320.91 mg kg^−1^ h^-^1. Respiration rate, being directly linked to metabolic activity, is regarded as a reliable indicator of fruit shelf-life [1,5,17]. The short shelf life of climacteric fruits is caused by an increase in respiration that occurs during the ripening process [6,16]. Tesfay and Magwaza [5] reported that in avocado, a common climacteric fruit, the rise in respiration rate caused by the increase of ethylene leads to a series of biochemical reactions that ultimately accelerate fruit softening.

In the current study, LDPE films successfully suppressed respiration rate in avocado fruit which can be attributed to the positive impact of packaging films in prolonging shelf life of perishable produce by delaying the processes of ripening and senescence through maintaining an ideal gaseous environment [12,17]. This supports our results as Fig. 3 shows that LDPE delayed fruit ripening by suppressing the rate of ethylene production of packaged fruit compared to control.

### 3.3 Weight loss

**Fig. 5:**
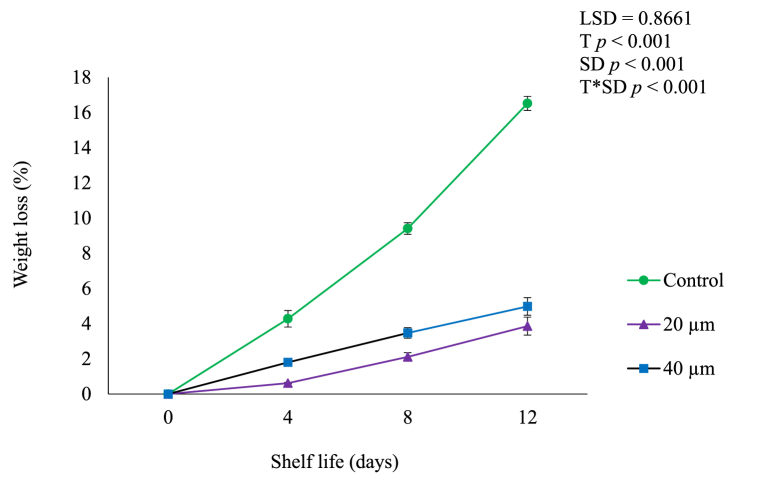
Weight loss of avocado fruit packed in LDPE films during shelf life of 12 days. Vertical bars represent standard error (SE) of the mean value (*n* = 3). The SE refers to the standard deviation of each measured values from the mean value. Means followed by different letters in each bar indicate a statistically significant difference (*p* < 0.001). LSD, least significant difference; T, treatments; SD, shelf life days; T*SD, treatments and shelf life days.

Most horticultural produce such as fruit and vegetables become unsaleable with moisture loss of 5–10% of their initial weight which causes quality degradation by promoting wilting and shriveling [9]. Extreme water loss, causes marketable weight loss, facilitates browning, loss of firmness and flavour, accelerated senescence, proneness to chilling-related damage and membrane and tissue breakdown of fruit [5,14]. Therefore, reducing moisture loss ensures economic profit and plays a crucial role in enhancing the storage period of fresh produce [9]. In this study, fruit weight loss significantly (*p* < 0.05) increased during shelf life of 12 days (Fig. 5) which could be attributed to the loss of moisture through transpiration, which is responsible for approximately 90% of the total weight loss in fruit [5]. Fruit moisture loss is primarily due to water pressure gradient or vapour pressure deficit between the less saturated ambient atmosphere and the fruit which is close to saturation with water [5,9]. However, fruit packed in 20 μm LDPE had the lowest weight loss of 3.86%, followed by fruit packed in 40 μm (4.98%) compared to unpacked fruit which had the highest weight loss of 16.52% at the end of shelf life. Lower weight loss of fruit in the LDPE packaging films could be attributed to slow rate of ripening indicated by reduced ethylene production and respiration rate (Fig. 3, Fig. 4) and prohibition of extreme moisture loss [26]. The comparatively lower water vapour transmission rate of LDPE packaging films may possibly contribute to the development of comparatively higher humidity within the package [9]. In addition, reduced weight loss in fruit packaged in LDPE plastic films could be attributed to the regulation of water vapour, oxygen and carbon dioxide transmission thus the decrease in respiration and transpiration rate whereas the increased weight loss in unpackaged fruit could be attributed to faster respiration and transpiration of the fruit [13,27]. This also supports our findings, Fig. 1, Fig. 2 shows that LDPE films modified gases within the package by decreasing O_2_ and increasing CO_2_ thus reducing fruit ethylene production (Fig. 3) and respiration rate (Fig. 4). This leads to delayed ripening thus the reduced weight loss in packed fruit in comparison to control [28]. These findings corroborate Kassim and Workneh [9], Hailu et al. [27] and Mohebbi et al. [29] who stated that LDPE plastics reduced moisture or weight loss in avocado, banana (*Musa* spp.) and cornelian cherry (*Cornus mas* L.) fruit during storage, respectively.

### 3.4 Firmness

**Fig. 6:**
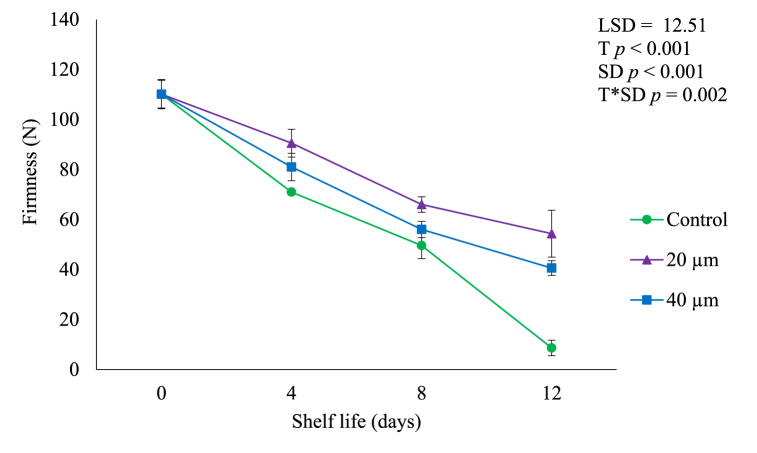
Firmness of avocado fruit packed in LDPE films during shelf life of 12 days. Vertical bars represent standard error (SE) of the mean value (*n* = 3). The SE refers to the standard deviation of each measured values from the mean value. Means followed by different letters in each bar indicate a statistically significant difference (*p* < 0.001). LSD, least significant difference; T, treatments; SD, shelf life days; T*SD, treatments and shelf life days.

In both 20 and 40 μm LDPE packaging films fruit firmness gradually (*p* < 0.05) decreased with increasing shelf life (Fig. 6). These trends were similar for both packaged and unpackaged fruit even though the rate of decline was different. Fruits from 20 μm LDPE bags were firmer (60.01 N) than fruit packed in 40 μm LDPE plastics (30.03 N) at the end of shelf life. This could be due to lower O_2_ and higher CO_2_ in 20 μm film compared to 40 μm bag which further reduces fruit metabolic activities [2,5]. The unpackaged fruit were the least firm (8.10 N) compared to the packaged fruit at the end of shelf life of 12 days. Reduced firmness loss in fruit packed in LDPE films could be due to the reduced ethylene production (Fig. 3) and respiration rate (Fig. 4) and weight loss (Fig. 5) [30]. In addition, loss of firmness in fruit during shelf life is associated with the movement of water from the peel to the pulp during ripening due to the osmosis process [24,31]. Low ethylene production in fruit packaged in LDPE films may result in lower cell wall enzyme activity and cell integrity preservation as cell wall enzymes are stimulated by ethylene which increases the firmness of the fruit [31]. Also, LDPE films with high CO_2_ (Fig. 2) prevent the breakdown of peptic substances which preserve fruit firmness and fruit remains firmer for an extended period [31].

Furthermore, loss of firmness or softening of fruit as the shelf life days progressed could be due to texture modification through deterioration of polysaccharides such as pectins, cellulose and hemicellulose that occurs during ripening [30]. Texture changes in fruits result from modifications by component polysaccharides which promote disassembly of primary cell wall and middle lamella structures due to enzyme activity on carbohydrate polymers [30,31]. Consequently, the variations in the reduction of firmness of avocado fruit packed in 20 and 40 μm films could possibly be attributed to the variations in the respiration rate that affect solubility and depolimerization of pectins during ripening [9,30].

### 3.5 Fruit size

Fruits have a comparatively high transpiration coefficient and water is lost rapidly from unpacked fruit leading to shrinkage or shriveling of the peel [16,28]. Table 1 shows that size of both packaged and unpackaged fruit decreased (*p* < 0.05) during shelf life of 12 days. However, fruit in 20 and 40 μm films maintained their size (23.91 and 23.40 mm) at the end of shelf life in comparison to control (22.11 mm). The decrease in fruit size could be due to shrinkage as a consequence of weight loss (Fig. 5) or loss of firmness (Fig. 6) which is linked to moisture evaporation and respiration through the exocarp. Therefore, LDPE packaging can retain a high humidity in the package to decrease transpiration or moisture loss thus weight loss (Fig. 5) and peel shrinkage or softening (Fig. 6) hence, maintaining fruit size.

**Table 1:** Effect of low-density polyethylene packaging films on physico-chemical properties of avocado during shelf life of 12 days.

SD	LDPE films (μm)	Fruit size (mm)	pH	Titratable acidity (mg.mL^−1^)	Soluble solid content (%)	SSC:TTA (%)
0	Control	24.21 ± 0.59 b	5.37 ± 0.38 a	20.54 ± 0.58 g	2.07 ± 0.08 ab	2.40 ± 0.22 a
4	Control	24.17 ± 0.04 b	5.53 ± 0.29 ab	16.73 ± 1.07 def	3.04 ± 0.04 c	5.02 ± 0.11 bc
8	Control	23.50 ± 0.22 b	5.97 ± 0.24 ab	12.70 ± 0.82 b	4.31 ± 0.40 ef	11.11 ± 0.47 de
12	Control	22.11 ± 0.06 a	6.04 ± 0.12 b	8.87 ± 0.44 a	6.86 ± 0.62 g	18.55 ± 1.44 f
0	20	24.21 ± 0.59 b	5.99 ± 0.21 bc	20.56 ± 0.59 g	2.00 ± 0.07 ab	2.41 ± 0.23 a
4	20	24.20 ± 0.02 b	6.79 ± 0.24 c	18.06 ± 1.04 efg	1.80 ± 0.45 a	6.41 ± 0.28 c
8	20	24.09 ± 0.05 b	6.82 ± 0.24 c	16.15 ± 1.56 cde	2.91 ± 0.15 bc	9.55 ± 1.19 d
12	20	23.91 ± 0.07 b	6.93 ± 0.20 c	14.43 ± 0.85 bcd	4.01 ± 0.35 de	11.08 ± 0.69 de
0	40	24.21 ± 0.59 b	6.11 ± 0.21 bc	20.53 ± 0.59 g	2.05 ± 0.03 ab	2.38 ± 0.22 a
4	40	24.19 ± 0.01 b	6.82 ± 0.09 c	19.22 ± 0.85 fg	2.66 ± 0.34 abc	4.04 ± 0.40 ab
8	40	24.01 ± 0.03 b	6.87 ± 0.09 c	17.11 ± 1.43 def	3.27 ± 0.26 cd	10.23 ± 0.88 d
12	40	23.40 ± 0.13 b	6.90 ± 0.06 c	13.39 ± 1.07 bc	5.01 ± 0.35 f	12.95 ± 1.38 e
		LSD = 1.036	LSD = 0.6323	LSD = 2.820	LSD = 0.9340	LSD = 1.900
		T *p* = 0.036	T *p* < 0.001	T *p* < 0.001	T *p* < 0.001	T *p* < 0.001
		SD *p* = 0.006	SD *p* = 0.016	SD *p* < 0.001	SD *p* < 0.001	SD *p* < 0.001
		T*SD *p* = 0.012	T*SD *p* < 0.001	T*SD *p* < 0.001	T*SD *p* = 0.014	T*SD *p* < 0.001

Values are the mean ± SE. Means within a column of the same parameter with different letters are significantly different according to Fisher's least significant difference test at *p* < 0.05. LSD, least significant difference; T, treatments; SD, shelf life days; T*SD, treatments and shelf life days; LDPE, low-density polyethylene; pH, potential of hydrogen; SSC, soluble solid content; TTA, titratable acidity.

### 3.6 pH

In this study, ‘Fuerte’ avocado fruit had pH values near neutrality as shown in Table 1. The pH values of both packaged and unpackaged fruit significantly (*p* < 0.05) increased during shelf life of 12 days. An increase in pH values can be linked with the conversion of organic acids and other complex compounds present in fruits into sugars which is a source of energy reserve utilized in the metabolic activities during ripening [21]. Fruit in 20 and 40 μm films had high (*p* < 0.05) pH values of 6.93 and 6.90 at the end of shelf life in comparison with unpackaged fruit (6.04). The higher pH values of packaged fruit could be attributed to the comparatively decreased ethylene production (Fig. 3) and respiration rate (Fig. 4) compared to unpacked fruit. Decreased O_2_ (Fig. 1) and increased CO_2_ (Fig. 2) which might be created due to the produce respiration could retard the rate of respiration (Fig. 4) in the package [29,32]. The lower pH of control fruit could be related to the production of acids through the breakdown of sugar at a rapid rate during shelf life [21]. The results observed this study are also in agreement with Mohebbi et al. [29] who stated that cornelian cherry fruit packed in LDPE films had high pH values during storage compared to control. Kassim and Workneh [9] reported that low pH values in “Hass” unpacked avocado could be attributed to quality degradation such as tissue damage in the fruit.

### 3.7 Titratable acidity

The titratable acidity (TTA) predicts the organic acid content of fruit which typically decreases during storage due to the use of organic acids as substrates for respiratory metabolism [28]. As shown in Table 1, TTA significantly (*p* < 0.05) decreased during shelf life in both packaged and unpackaged fruit, however, this decrease was low in fruit packed in LDPE films. Fruit in 20 and 40 μm LDPE films had high TTA values of 14.43 and 13.39 mg mL^−1^ at the end of shelf life compared to unpackaged fruit which had lower TTA (8.87 mg mL^−1^). The TTA preservation by LDPE films may be due to a retardation in the ripening and maturation processes, which subsequently decreases the metabolic activities leading to TTA degradation [7,29]. The atmospheric modification created when fruit are packaged in LDPE bags could retard respiration and as a direct effect, the consumption of respiration substrates including organic acids and sugars is inhibited [29]. Therefore, as the fruit respires, the O_2_ concentration may decrease (Fig. 1) and the CO_2_ concentration increases (Fig. 2) in the LDPE films [31]. Conversely, the increased TTA loss observed in unpackaged fruit can be attributed to a decline of organic acids due to a rapid respiration and ripening rate of fruit during ambient storage [27,29,31].

### 3.8 Soluble solid content

As presented in Table 1, soluble solid content (SSC) of both packed and unpacked avocado fruit progressively (*p* < 0.05) increased during shelf life. However, fruit packed in 20 and 40 μm LDPE films had low TSS of 4.01 and 5.01% at the end of shelf life in comparison with control which had higher TSS content of 6.86%. Generally, packaging avocado fruit in LDPE bags showed better conservation of SSC content during shelf life compared to control. The possible atmospheric alteration, particularly reduced O_2_ (Fig. 1) and increased CO_2_ (Fig. 2) created in the package may have delayed ripening of the fruit due to reduced respiration rate [9,33]. Thus, packaged fruit do not quickly deplete their soluble solids as those of the unpacked ones [33] as found in present study.

In addition, reduced O_2_ in packaging materials slows down the ethylene biosynthesis and subsequent changes in fruit texture [31,34]. Hence, LDPE films can enhance fruit quality by reducing O_2_ availability (Fig. 1) and modifying internal gas composition, reducing oxidative metabolism and retarding textural changes in packaged fruit [9]. Our findings are similar to Kassim and Workneh [9] and Mohebbi et al. [29] who found that LDPE films effectively maintained TSS of cornelian cherry and avocado during storage.

### 3.9 Sugar:acid ratio

The SSC/TTA also known as the ripening index [28] significantly (*p* < 0.05) increased in both packaged and unpackaged fruit (Table 1). This increase was significantly (*p* < 0.05) higher in control than in fruit packed in LDPE films. At the end of shelf life, fruit packaged in both 20 and 40 μm films had lower SSC/TTA (11.08 and 12.95%) compared to control which had high ripening index (18.55%). Ripening index increases as a consequence of conversion of complex carbohydrates (starch) into simple carbohydrates and loss of titratable acidity [28]. A higher ripening index value is an indication of an advanced stage of fruit ripening [35]. In our study, LDPE films modified gas atmosphere inside the package through reduced O_2_ (Fig. 1) and increased CO_2_ (Fig. 2) created in the package thus, delaying starch hydrolysis and maintaining higher TTA level (Table 1), consequently keeping the value of ripening index lower [9,28]. In this study, fruit packaged in LDPE films had lower ripening index than control due to low conversion of starch into sugars (Table 1). Overall, LDPE films preserved the quality of avocado fruit during shelf life by reducing physiological weight loss (Fig. 5) and starch hydrolysis and preserving TTA and ripening index (Table 1). Our findings are similar to Mohebbi et al. [29] who stated that unpackaged cornelian cherry had higher SSC/TTA compared to the ones in LPDE films due to both lower titratable acidity and higher weight loss, resulting in the condensation of SSC.

### 3.10 Oxidative stress

**Fig. 7:**
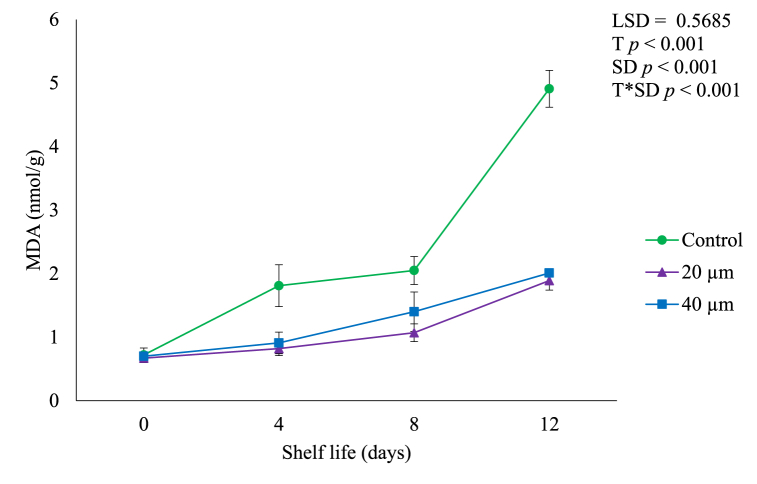
Malondialdehyde (MDA) content of avocado fruit packed in LDPE films during shelf life of 12 days. Vertical bars represent standard error (SE) of the mean value (*n* = 3). The SE refers to the standard deviation of each measured values from the mean value. Means followed by different letters in each bar indicate a statistically significant difference (*p* < 0.001). LSD, least significant difference; T, treatments; SD, shelf life days; T*SD, treatments and shelf life days.

The MDA content and LOX activity are used as measurements of membrane integrity loss in reaction to postharvest oxidative stress during shelf life of horticultural produce [5]. The change of MDA level is regarded as a marker of membrane lipid peroxidation of fruits subjected to senescence or stress [34]. From Fig. 7, MDA contents of unpackaged fruit significantly (*p* < 0.05) increased (0.71–4.81 nmol/g) at a faster rate during shelf life compared to fruit packed in 20 μm (0.68–1.89 nmol/g) and 40 μm (0.70–2.01 nmol/g) films. Overall, fruit in LDPE films had low MDA contents compared to control. Tesfay and Magwaza [5] reported that the oxidation of polyunsaturated fatty acids leads to oxidants including peroxide ions and MDA. Also, high fruit lipid peroxidation and accumulation of MDA is associated with the ripening processes and increases with the advancing senescence of fruit [34,36]. In our study, the high accumulation of MDA is control than fruit in LDPE films is probably due primarily to postharvest ripening as observed that control fruit had low pH and TTA and high SSC and SSC/TTA (Table 1) [36]. This increase could also be attributed to the senescence progress in which control fruit show significant quality deterioration particularly high weight loss (Fig. 5) and softening (Fig. 6) [29].

**Fig. 8:**
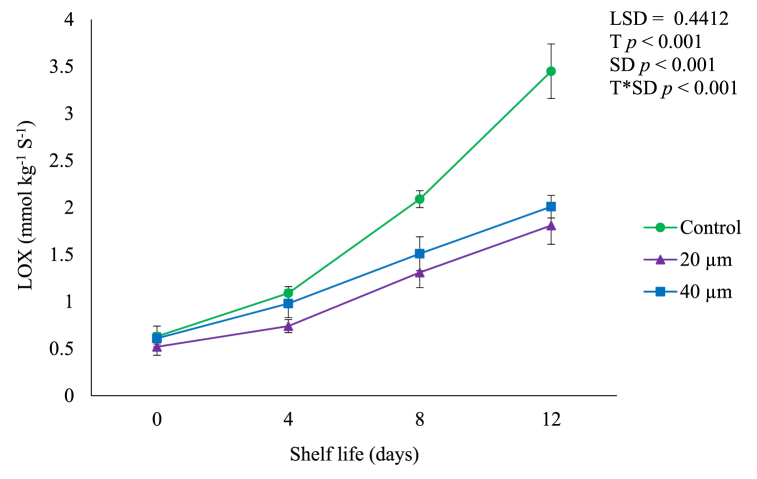
Lipoxygenase (LOX) activity of avocado fruit packed in LDPE films during shelf life of 12 days. Vertical bars represent standard error (SE) of the mean value (*n* = 3). The SE refers to the standard deviation of each measured values from the mean value. Means followed by different letters in each bar indicate a statistically significant difference (*p* < 0.001). LSD, least significant difference; T, treatments; SD, shelf life days; T*SD, treatments and shelf life days.

A similar trend was noted for LOX activity where a significant (*p* < 0.05) increase was observed during shelf (Fig. 8) due to dioxygenation of polyunsaturated fatty acids producing toxic hydroperoxy fatty acids and consequent membrane damage [34]. Fruit in 20 and 40 μm LDPE films showed lower LOX activity of 1.81 and 2.00 mmol kg^−1^ S^−1^ at the end of shelf life compared to control (3.36 mmol kg^−1^ S^−1^), implying greater conservation of membrane integrity [29,36]. Fig. 7 shows that fruit packed in LDPE films resulted in low MDA which reflect maintenance of membrane integrity [36].

Overall, the results of the current study demonstrated that LDPE films effectively decreased accumulation of MDA content and LOX activity (Fig. 7, Fig. 8). Therefore, LDPE films can be an effective tool for inhibiting oxidative damage and maintaining membrane integrity through modification of the gaseous composition within the packaging [32]. Nath et al. [37] and Pan and Sasanatayart [38] observed a rapid increase in CO_2_ levels and decrease in O_2_ levels inside the LDPE packed pear (*Pyrus communis* L.) and fresh-cut bok choy (*Brassica rapa* var. chinensis) during initial storage at ambient conditions due to reduced respiration rate. Minimum MDA contents and LOX activity in fruit packed in LDPE films may possibly be due to less availability of O_2_ (Fig. 1) for respiration which eventually inhibited respiration rate (Fig. 4) and thus reducing quality deterioration such as the weight loss (Fig. 5) or firmness loss (Fig. 6) due to transpiration, thus, preventing lipid oxidation such as MDA (Fig. 7) and LOX activity (Fig. 8) and extending fruit shelf life [7,38].

### 3.11 Fruit decay

Table 2 shows that control fruit had severe (*p* < 0.05) stem end rot, side rot (alternaria rot and anthracnose) and overall decay at the end of shelf life. Fruit packed in 20 μm LDPE films had no decay incidence whereas those in 40 μm has slight symptoms of decay at the end of shelf life. This can be attributed to lower respiration and ethylene production rates which retarded ripening and senescence, hence inhibiting the growth of decay causing pathogens due to modification of the gas atmosphere inside the package, thus, preserving quality and prolonging shelf life of fruit [16,17]. This further supports our findings as fruit packed in LDPE films had reduced ethylene production and respirations rates (Fig. 3, Fig. 4), high pH and TTA and low SSC (Table 1) indicating that LDPE films delayed fruit ripening during shelf life compared to control. Furthermore, LDPE films maintained fruit quality as indicated by reduced weight loss and firmness loss (Fig. 5, Fig. 6) during shelf life compared to control. Our findings are similar to Mohebbi et al. [29] who stated that cornelian cherry packed in LDPE films had no symptoms of decay during storage of 35 days compared to control which could be attributed to modified gas atmosphere in the package such as a rapid increase in CO_2_ levels and decrease in O_2_ levels in the LDPE plastic packed cornelian cherry during initial storage due to reduced respiration rate of fruit.

**Table 2:** Decay incidence of avocado packaged in LDPE films and control fruit during shelf life of 12 days.

SD	LDPE films (μm)	Stem-end rot	Side rot (alternaria rot and anthracnose)	Overall decay
0	Control	1.00 ± 0.00 a	1.00 ± 0.00 a	1.00 ± 0.00 a
4	Control	1.00 ± 0.00 a	1.00 ± 0.00 a	1.00 ± 0.00 a
8	Control	2.33 ± 0.33 c	2.67 ± 0.33 b	3.00 ± 0.65 c
12	Control	5.00 ± 0.53 d	5.00 ± 0.48 c	5.00 ± 0.87 d
0	20	1.00 ± 0.00 a	1.00 ± 0.00 a	1.00 ± 0.00 a
4	20	1.00 ± 0.00 a	1.00 ± 0.00 a	1.00 ± 0.00 a
8	20	1.00 ± 0.00 a	1.00 ± 0.00 a	1.00 ± 0.00 a
12	20	1.00 ± 0.00 a	1.00 ± 0.00 a	1.00 ± 0.00 a
0	40	1.00 ± 0.00 a	1.00 ± 0.00 a	1.00 ± 0.00 a
4	40	1.00 ± 0.00 a	1.00 ± 0.00 a	1.00 ± 0.00 a
8	40	1.33 ± 0.33 ab	1.33 ± 0.33 a	1.33 ± 0.33 a
12	40	1.67 ± 0.37 b	1.33 ± 0.33 a	2.00 ± 0.58 b
	LSD =	LSD = 0.4582	LSD = 0.5034	LSD = 0.5449
	T *p*	T *p* < 0.001	T *p* < 0.001	T *p* < 0.001
	SD *p*	SD *p* < 0.001	SD *p* < 0.001	SD *p* < 0.001
	T*SD *p*	T*SD *p* < 0.001	T*SD *p* < 0.001	T*SD *p* < 0.001

Values are the mean ± SE. Means within a column of the same parameter with different letters are significantly different according to Fisher's least significant difference test at *p* < 0.05. LSD, least significant difference; T, treatments; SD, shelf life days; T*SD, treatments and shelf life days; LDPE, low-density polyethylene.

### 3.12 Percentage marketability

**Fig. 9:**
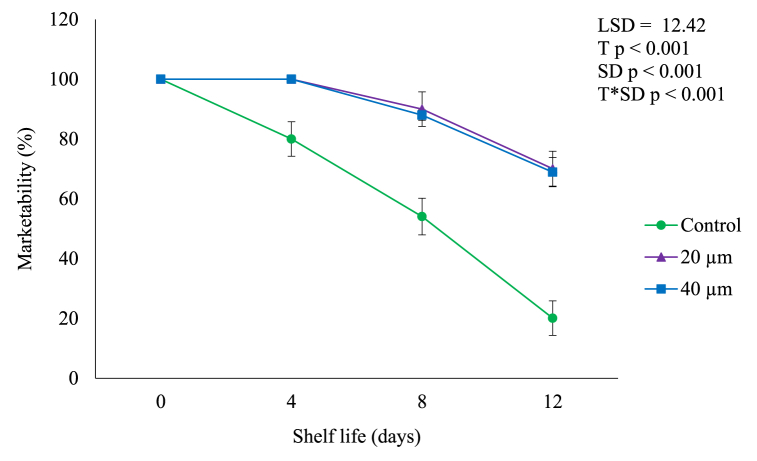
Marketability of avocado fruit packed in LDPE films during shelf life of 12 days. Vertical bars represent standard error (SE) of the mean value (*n* = 3). The SE refers to the standard deviation of each measured values from the mean value. Means followed by different letters in each bar indicate a statistically significant difference (*p* < 0.001). LSD, least significant difference; T, treatments; SD, shelf life days; T*SD, treatments and shelf life days.

The percentage of fruit marketability gradually (*p* < 0.05) decreased during shelf life (Fig. 9). However, LDPE films had a significant (*p* < 0.05) effect on the percentage marketability of avocado fruit. The percentage marketability of fruit packed in 20 and 40 μm LDPE films decreased from 100.00 to 70.41% and 100–68.01% during shelf life of 12 days. Therefore, fruit packed in LDPE films were markable (>65%) at the end of shelf life. This can be attributed to the modified atmosphere created inside the package, for instance, reduced O_2_ (Fig. 1) and increased CO_2_ (Fig. 2) within the packaging films decreases the respiration and transpiration rate of fruit [29]. In our study, LDPE films reduced ethylene production and respiration rate (Fig. 3, Fig. 4), weight loss (Fig. 5), softening (Fig. 6) and shrinkage of fruit (Table 1). Moreover, packaged fruit had no symptoms of decay or reduced decay incidence (Table 2) during shelf life. In addition, the difference in the percentage marketability between the films could be associated with the permeability difference between the two plastic films for water vapour and gases [29].

On the other hand, the percentage marketability of control fruit significantly (*p* < 0.05) decreased from 100 to 20.01% during shelf life. Therefore, unpacked fruit were unmarkable (20.01%) at the end of shelf life of 12 days. The termination of shelf life of avocado kept at ambient conditions is determined by shriveling, over ripening, discoloration and mold growth [9]. In this study, control fruit had high ethylene production and respiration rates (Fig. 3, Fig. 4) which accelerated ripening process in fruit. Faster transpiration rate may result in fruit weight loss (Fig. 5), softening (Fig. 6), shriveling of fruit (Table 1). Moreover, higher respiration rate may lead to senescence of fruit as Table 2 shows that control fruit had severe symptoms of decays at the end of shelf life, thus unmarketable (Fig. 9).

## 4 Conclusion

The use of low-density polyethylene (LDPE) packaging materials to preserve postharvest quality and prolong shelf life of avocado fruit may be regarded as an effective and alternative technique of fresh avocado fruit storage at ambient conditions. When avocado fruit were packaged in LDPE bags, a modified atmosphere was created, where the O_2_ levels decreased and the CO_2_ levels increased in the bags as the fruit ripen. The LDPE films effectively delayed ripening of fruit by reducing ethylene production and respirations rates and fruit had high pH, titratable acidity and low soluble solid content and sugar:acid ratio as a result of delayed ripening during shelf life compared to control. Fruits packaged in LDPE plastics retained better fresh weight and retained firmness during the storage period compared to unpackaged fruit. In addition, packaged fruit had low MDA and LOX activity, no decay incidence (20 μm film) or slight symptoms of decay (40 μm film) and were markable throughout the shelf life of 12 days compared to control which had high levels of lipid oxidation or oxidative stress, severe decay symptoms and were unmarketable at the end of shelf life days. Although sensory evaluation showed that the packaging films were effective in controlling fruit decay during shelf life, future studies should further investigate the effectiveness of LDPE on controlling fungal pathogens in fruit. Future studies should also investigate the different manufacturing processes and parameters which can lead to significant differences in material properties and postharvest quality of horticultural produce.

## Data availability

Data will be made available on request.

## Ethics statement

Fruit decay evaluation and the percentage of marketability studies were conducted according to established ethical guidelines, and informed consent obtained from the participants.

## Additional information

No additional information is available for this paper.

## CRediT authorship contribution statement

**Nana Millicent Duduzile Buthelezi:** Writing – review & editing, Writing – original draft, Visualization, Validation, Supervision, Software, Resources, Project administration, Methodology, Investigation, Funding acquisition, Formal analysis, Data curation, Conceptualization. **Tieho Paulus Mafeo:** Writing – review & editing.

## Declaration of competing interest

The authors declare that they have no known competing financial interests or personal relationships that could have appeared to influence the work reported in this paper.
